# *Trichomonas vaginalis* as a risk factor for human papillomavirus: a study with women undergoing cervical cancer screening in a northeast region of Brazil

**DOI:** 10.1186/s12905-021-01320-6

**Published:** 2021-04-23

**Authors:** Ilka Kassandra Pereira Belfort, Ana Paula Almeida Cunha, Francisco Pedro Belfort Mendes, Leonardo Victor Galvão-Moreira, Renata Gaspar Lemos, Lucas Henrique de Lima Costa, Pablo Monteiro, Mariele Borges Ferreira, Gerusinete Rodrigues Bastos dos Santos, Joyce Leal Costa, Alice de Sá Ferreira, Luiz Gustavo Oliveira Brito, Luciane Maria Oliveira Brito, Flávia Castello Branco Vidal, Sally Cristina Moutinho Monteiro

**Affiliations:** 1 Doctoral Program in Biotechnology - Northeast Biotechnology Network (RENORBIO), Federal University of Maranhão (UFMA), Rua 4 Quadra 9 Casa 3 Residencial Primavera, São Luis, MA 65052-850 Brazil; 2 Postgraduate Program in Adult Health, Federal University of Maranhão, São Luís, MA Brazil; 3 Federal University of Maranhão (UFMA), São Luís, MA Brazil; 4 Department of Pharmacy, Federal University of Maranhão (UFMA), São Luís, MA Brazil; 5 Department of Tocogynecology (DTG), Faculty of Medical Sciences (FCM), State University of Campinas (UNICAMP), Campinas, Brazil

**Keywords:** Sexually transmitted infection, *Trichomonas vaginalis*, Human papillomavirus, Co-infection

## Abstract

**Background:**

Human papillomavirus (HPV) and *Trichomonas vaginalis* (TV) infections are the most common sexually transmitted infections (STIs) globally. The latter has contributed to a variety of adverse outcomes for both sexes. Moreover, in Brazil, epidemiological studies on patients with STIs are limited. Therefore, this study aimed to determine the prevalence of TV and its association with HPV in women undergoing cervical cancer screening.

**Methods:**

Women with a normal cervix were recruited from a community-based cervical cancer screening program. Gynecological examinations were conducted, and questionnaires were provided. Vaginal canal and uterine cervix samples were collected for cytological examinations (reported using the 2001 Bethesda System) and tested for the presence of TV and HPV DNA.

**Results:**

In total, 562 women who attended public primary healthcare were included in the study. The *T. vaginalis* was present in 19.0% (107) and HPV DNA was present in 46.8% (263) of women. Among the women of TV 73.8% (79) had a co-infection with HPV (*p* = 0.001).

**Conclusions:**

We concluded that a TV infection is associated with an HPV infection of the cervix as well as with the cervical cytological abnormalities. Further studies could reveal the mechanisms by which these two organisms interact at the cellular level, with control for shared behavioral risk factors.

## Background

Sexually transmitted infections (STIs) represent a significant public issue that affects people`s health worldwide. [1]. In 2016, an estimation of 376 million new infections (more than 1 million per day) was reported in people aged between 15 and 49 years [2]. *Trichomonas vaginalis* (TV) and Human Papillomavirus (HPV) infections are among the most common STIs; however, the prevalence of these STIs varies significantly globally, with a higher prevalence in countries with low socioeconomic indexes [3].

These infections can lead to multiple complications, especially in women, including urogenital-related issues (cervicitis, urethritis, vaginitis, and genital ulceration), complications during pregnancy, infertility, increased risk of acquisition and transmission of human immunodeficiency virus, and cancer [3–5].

HPV is associated with several types of cancer, including cervical and penile cancer [6]. The incidence of cervical cancer is higher than 528,000 cases per year, with more than 270,000 deaths caused by it [1]. The global prevalence of HPV in women with normal cytology is estimated at 11.7% but can reach up to 55.4% in Brazil [1, 7].

Persistent infection by viral types of high oncogenic risk, mainly by HPV types 16 and 18, is one of the main factors for the development of cervical cancer. Many co-factors contribute to the persistent HPV infection and the progression of cervical lesions, including biological, behavioral, and environmental factors, as well as other sexually transmitted infections (STIs) [8].

Sexually transmitted infections may play a crucial role in HPV persistence, leading to serious complications, since it facilitates HPV entry (single or multiple viruses) and decreases the host´s ability to resolve the viral infection. Moreover, the chronic inflammation caused by STI and HPV can act as a promoter of carcinogenesis by inducing the occurrence of significant cellular and molecular changes [9–11].

*Trichomonas vaginalis* (TV) is a flagellated, facultatively anaerobic protozoan of the human genital tract [12]. It is the most common, non-viral, sexually transmitted agent worldwide, responsible for 143 million cases in 2012 and 110.4 million in 2018 [2]. Epidemiological studies have shown that TV infection can lead to an increased risk of cervical cancer [13–16]. The interaction between cervical cancer and TV is not yet fully elucidated, but it is believed that the inflammatory process caused by this protozoan predisposes the epithelium to carcinogenesis [12, 17–19]. Cervical epithelium disruption is due to the inflammation process caused by TV, which facilitates the entry of HPV into the basal layer of the epithelium. As a result, it leads to the integration of viral DNA into the host DNA and the overexpression of viral oncogenes that contribute to the activation of carcinogenic mechanisms [17, 19, 20].

In this perspective, the study goal was to determine the prevalence of TV and its association with HPV in women who sought to undergo cervical cancer screening in Northeastern Brazil.

## Methods

### Study population

This cross-sectional and non-interventional study was conducted with 562 women who were patients at the public health units of gynecological care in São Luís, Maranhão, Brazil. The sample size was calculated considering a prevalence of 11.2% of women with TV [2], a power of 90% and a 5% significance level. The calculated sample was 384 women; however, 562 women were included in the study.

The study was approved by the Ethics Committee on Human Research (Federal University of Maranhão - protocol number 2.383.604) and all the potential women were counselled by the investigators about the objectives and methodology of the study. Was obtained written informed consent from all participants. Consent forms were kept separately from questionnaires and biological samples so names could not be linked to any study data collected.

Sociodemographic and clinical data were obtained by semi structured questionnaire based on validated instruments (age, ethnicity, education, family income, professional activity, marital status, sexual behavior, alcohol consumption, smoking status, and reproductive health and barrier methods used). The following exclusion criteria were applied: women who were menstruating, underwent hysterectomy, were virgins, or pregnant for less than 45 days postpartum.

### Cervical cytology

Ayre’s spatula was used to obtain cervical scraping, fixed on a glass slide with ethanol, and stained using for Pap smear. Cytological examinations of Pap smear were reported using the 2001 Bethesda Reporting System.

### Specimen collection and DNA extraction

For HPV DNA isolation, samples were obtained using a Digene HC2 DNA Collection Device (QIAGEN, CA, USA) and stored at − 20 °C until processed. DNA extraction was done using the QIAamp DNA Mini and Blood kit (QIAGEN, CA, USA), according to the manufacturer instructions. DNA extraction was confirmed by amplification of the β-globin gene. Total DNA was isolated, eluted in 100 mL AE buffer, and stored at − 20 °C.

### HPV and TV detection

The presence of HPV DNA was detected using nested polymerase chain reaction (PCR) with the primer sets PGMY09/11 (first round of PCR) and GP+5/GP+6 (second round of PCR) [21]. Presence of TV was detected using conventional PCR with the primers TVA5/6 [22].

Amplification products were evaluated using electrophoresis with a 1.5% agarose gel in 1× TBE buffer for 30 minutes at 5 V/cm in a horizontal unit (Life Technologies, Carlsbad, CA, USA). Bands were stained with 0.1% Gel Red (Invitrogen) and visualized using an ultraviolet transilluminator (BioRad Laboratories, Hercules, CA, USA).

### HPV Genotyping

PCR products were typed by Big Dye Terminator v3.1 Cycle Sequencing Kit (Applied Biosystems, Foster City, CA), and analyzed using an ABI Prism 3130XL Genetic Analyzer (Applied Biosystems). The sequences were edited and analyzed using 4Peaks Software (Nucleobytes, Amsterdam, Netherlands). HPV genotypes were identified using the BLASTn (Basic Local Alignment Search Tool, http://blast.ncbi.nlm.nih.gov/).

### Data analysis

Statistical analyses were performed using the IBM SPSS® software version 23. Initial descriptive analyses were performed to assess the categorical variables, and the chi-square test was performed for comparison between groups. The results were considered statically significant when *p* ≤ 0.05. After a univariate analysis performed by the chi-square test, variables with a significance level ≤ of 0.05 were selected to assess their independent contribution to the onset of trichomoniasis infection through a multivariate analysis using binary logistic regression. In addition, a ROC (Receiver Operating Characteristic) curve was performed to verify the quality of the prediction of the final regression model.

## Results

In total, 562 women who attended public primary healthcare from June 2017 to July 2019 were included in the study. *Trichomonas vaginalis* was present in 19.0% (107), and HPV DNA was present in 46.8% (263) of women. Among women of TV, 73.8% (79) had a co-infection with HPV (*p* = 0.001).

Women aged between 30 and 49 years (48.40%), self-declared non-white color (90.04%), no having a fixed partner (52.49%), and women with high-school level education (52.49%) were predominant. Regarding risk factors and sexual habits, most women reported non-smokers (90.39%), non-alcoholics (62.10%), not using condoms during sexual intercourse (77.05%), having anal (67.97%) and oral sex (95.55%), and absence of previous STI (60.85%).

The results of cytology testing reported that 48 (8.5%) women had cervical abnormalities, which included 16 (2.8%) classified as ASCUS (Atypical Squamous Cells of Unknown Significance), 6 (1.1%) classified as ASCH (Atypical Squamous Cells of Indeterminate Significance), 16 (2.8%) classified as LGSIL (Low-Grade Squamous Intraepithelial Lesion), 4 (0.7%) classified as HGSIL (High-grade squamous intraepithelial lesion), and 6 (1.1%) classified CIN II + CIN III (Cervical Intraepithelial Neoplasia) (Table 1).

**Table 1.** Demographic, sexual habits and clinical data of woman treated in public health care, São Luís, Maranhão, Brazil

	Total (N = 562)		*Trichomonas vaginalis*				*p* value
			Yes (N = 107)		No (N = 455)		
	N	%	N	%	N	%	
*Age*							
< 29 years	155	27.58	30	19.35	125	80.65	0.473
30–49 years	272	48.40	56	20.59	216	79.41	
50+ years	135	24.02	21	15.56	114	84.44	
*Skin color*							
White	56	9.96	5	8.93	51	91.07	**0.042**
Non-white	506	90.04	102	20.16	404	79.84	
*Relationship status*							
With partner	267	47.51	51	19.10	216	80.90	0.972
Single	295	52.49	56	18.98	239	81.02	
*Education level*							
Elementary school	196	34.88	41	20.92	155	79.08	0.707
High-school level	295	52.49	53	17.97	242	82.03	
Graduate school	71	12.63	13	18.31	58	81.69	
*Smoking status*							
No	508	90.39	95	18.70	413	81.30	0.531
Yes	54	9.61	12	22.22	42	77.78	
*Alcohol consumption*							
No	349	62.10	65	18.62	284	81.38	0.749
Yes	213	37.90	42	19.72	171	80.28	
*Condom use*							
No	433	77.05	73	16.86	360	83.14	**0.016**
Yes	129	22.95	34	26.36	95	73.64	
*Menarche*							
Before 13 years old	342	60.9	52	48.6	290	63.7	**0.004**
After 13 years old	220	39.1	55	51.4	165	36.3	
*Anal sex*							
No	180	32.03	38	21.11	142	78.89	0.390
Yes	382	67.97	69	18.06	313	81.94	
*Oral sex*							
No	25	4.45	3	12.00	22	88.00	0.359
Yes	537	95.55	104	19.37	433	80.63	
*Cytological abnormality*							
No	514	91.5	86	80.4	428	94.1	**0.001**
ASC-US	16	2.8	6	5.6	10	2.2	
LGSIL	16	2.8	8	7.5	8	1.8	
ASC-H	6	1.1	4	3.7	2	0.4	
HGSIL	4	0.7	3	2.8	1	0.2	
CIN II + CIN III	6	1.1	0	0	6	1.3	
*HPV*							
No	299	53.2	28	26.2	271	59.6	**0.001**
Yes	263	46.8	79	73.8	184	40.4	

Bold values indicate the results were considered statically significant when *p* ≤ 0.05

*ASCUS* atypical squamous cells of unknown significance, *ASCH* atypical squamous cells of indeterminate significance, *LGSIL* Low-Grade Squamous Intraepithelial Lesion, *HGSIL* high-grade squamous intraepithelial lesion, *CIN II* cervical intraepithelial neoplasia II, *CIN* cervical intraepithelial neoplasia III

Infection with TV was associated with skin color (*p* = 0.042), not using condoms (*p* = 0.016), age of menarche (*p* = 0.004), presence of cytological abnormalities (*p* = 0.001), and HPV infection (*p* < 0.001) (Table 1).

The univariate analysis reported statistically significant results (*p* < 0.05) among the variables color, no condom use, cytological alteration, HPV infection, and menarche. Thus, we performed a binary logistic regression model to verify the prediction of these explanatory variables regarding the acquisition of *Trichomonas vaginalis* infection.

The final regression model was constructed using all the significant variables from the univariate analysis in block 1. The variables that showed *p* ≤ 0.2 were included in block 2 (menarche, no condom use, cytological alteration, and HPV infection). All these variables showed *p* ≤ 0.05 by Wald's statistics, so they remained in the final model.

Binary logistic regression showed that menarche remained associated with TV infection, and women who had menarche after 13 years of age are 2111 more likely to contract TV.

Similarly, HPV is also associated with trichomoniasis in a multivariate analysis, showing that HPV infection increases by 2297 a chance of developing TV infection.

On the other hand, the use of condoms is a protective factor against the non-development of TV, corroborating the univariate analysis, where the non-use of condoms resulted in a relationship with TV. Thus, the use of condoms decreases by 0.577 times the chance of acquiring TV infection.

Also, when verifying the association between cytological alteration and TV, the binary logistic regression model showed that women who had LGSIL and ASCH infection have 3179 and 12047 more chances of having TV infection, respectively. Table 2 shows the multivariate analysis using binary logistic regression.

**Table 2.** Multivariate analysis using binary logistic regression for the infection of *Trichomonas vaginalis* of woman treated in public health care, São Luís, Maranhão, Brazil

	*Trichomonas vaginalis*		*p* value
	*Odds ratio -IC (95%)*	*Wald*	
*Menarche*			
Before 13 years old	Ref		
After 13 years old	2.111 (1.328–3.356)	9.987	**0.002**
*Use condon*			
Yes	0.577 (0.348–0.956)	4.548	**0.033**
No	Ref		
*HPV*			
Yes	2.297 (1.461–3.610)	12.993	**0.001**
No	Ref		
*Color*			
White	Ref		
Nonwhite	2.216 (0.844–5.815)	2.613	0.106
*Cytological abnormality*			
No	Ref	–	–
ASC-US	2.659 (0.878–8.052)	2.993	0.084
LGSIL	3.179 (1.099–9.201)	4.552	**0.033**
ASC-H	12.047 (1.986–73.069)	7.323	**0.007**
HGSIL	8.167 (0.815–81.872)	3.189	0.074
*CIN II + CIN III*	0	0	0

Bold values indicate the results were considered statically significant when *p* ≤ 0.05

*ASCUS* atypical squamous cells of unknown significance, *ASCH* atypical squamous cells of indeterminate significance, *LGSIL* low-grade squamous intraepithelial lesion, *HGSIL* high-grade squamous intraepithelial lesion, *CIN II* cervical intraepithelial neoplasia II, *CIN* cervical intraepithelial neoplasia III

**Fig. 1:**
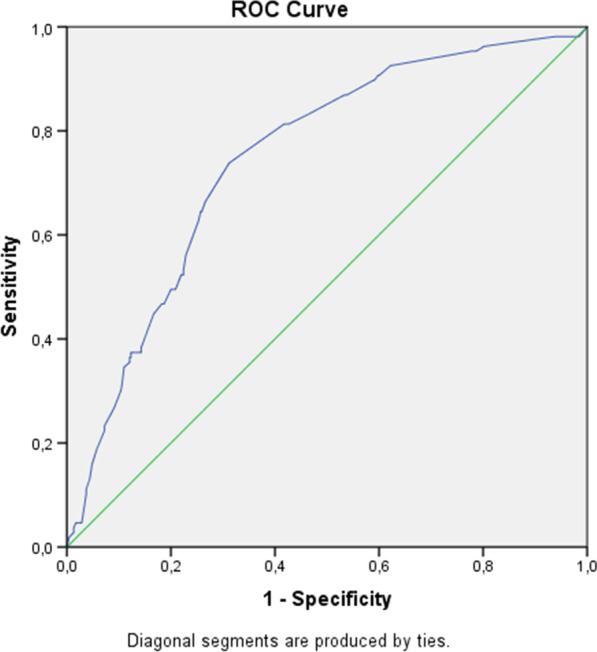
Discriminatory power of the binary logistic regression model for *Trichomonas vaginalis* infection of woman treated in public health care, São Luís, Maranhão, Brazil

We used the ROC curve (Receiver Operating Characteristic) to verify the fit quality of the final model regarding the discriminatory power of TV infection. The area under the curve showed a value of 0.746, considered a good discrimination power (Fig. 1).

HPV genotyping was performed in 263 samples, of which showed that 85 (32.3%) of cervical samples were classified as high risk, 40 (15.2%) were classified as low risk and 138 (52.4%) samples were classified as indeterminate (low or high risk). We performed a binary logistic regression to evaluate the relationship between HPV viral types and TV infection. However, the 138 undetermined genotypes were excluded from the analysis, as it would behave as a possible confounding factor in the analysis.

In the regression analysis, no association between the specific kinds of HPV and TV was found, and it was possible to verify that not having HPV infection decreases the chance of having TV by 0.272 times. Data presented in Table 3.

**Table 3.** Multivariate analysis using binary logistic regression for the development of *Trichomonas vaginalis* in woman treated in public health care, São Luís, Maranhão, regarding HPV genotyping

	Odds ratio-IC (95%)	Wald	*p* value
*HPV*			
Negative	0.272 (0.159–0.466)	22.399	**0.000**
High risk	1.096 (0.603–1.993)	0.091	0.763
Low risk	1.754 (0.842–3.657)	2.249	0.134

Bold value indicate the results were considered statically significant when *p* ≤ 0.05

## Discussion

HPV and TV infections are among the most common STIs worldwide, both associated with multiple health consequences in men and women [12]. TV infection can lead to an increased risk of cervical cancer [13–16]. The interaction between cervical cancer and TV is not yet fully elucidated, but it is believed that the inflammatory process caused by this protozoan predisposes the epithelium to carcinogenesis [12, 17–19].

Studies have shown that a previous history of infection with TV leads to an increased risk of HPV infection, mainly owing to the viral types of high oncogenic risk [13, 18, 23]. HPV is considered the etiological factor of cervical cancer; however, the fact that some women manage to eliminate HPV without the development of cervical lesions leads to the question that other cofactors can facilitate the persistence of this virus, thereby preventing its elimination and favoring cervical changes mediated by HPV [18].

In this context, the present study affirmed the association of TV with HPV. It was thus demonstrated that HPV is a risk factor for TV, suggesting that there is a possible cooperation between both microorganisms, contributing to cellular microenvironment changes.

TV releases lytic enzymes that reduce the protective mucus layer of the vaginal wall, leading to a reduction in vaginal fluids [13]. This can lead to the development of micro lesions in the epithelium, thereby increasing virulence the HPV and allowing the integration of the DNA into the host cell, which can lead to host cell DNA damage and the beginning of the carcinogenic process [12]. The inflammatory process can also rupture the basal layer of the cervical epithelium and thus facilitate its persistence in the cervical-vaginal epithelium tissue [12, 19].

In this context, it is important to evaluate the influence of TV and HPV co-infection in the genital tract of women without cervical cancer to understand these cofactors. However, the differences in prevalence are observed globally, with 1.9% in Busan/South Korea [24], 3.1% in Shanghai/China [25], 5.6% in female sex workers in the Midwest region of Brazil [26], 5.7% in Bahia/Brazil [27], 31.4% in Kenya [28], 18.8% in Beijing/China [29], and 24% in the rural area of Ngaramtoni /Tanzania [13]. The results presented here demonstrate a 27% co-infection prevalence of TV and HPV, which is in line with the studies mentioned above.

In addition to HPV infection, multivariate analysis showed that other cofactors were also associated with TV infection in the study population, such as cytological abnormality and inconsistent condom use. Studies have found an association between TV infection, cervicitis, and vaginal infections in the increased risk of squamous intraepithelial lesions and/or cervical intraepithelial neoplasia (CIN) [28–30], mainly associated with persistent HPV infection [25, 30–32].

The mechanism associated with cervical dysplasia and persistent HPV infection in the context of cohabitating STIs is the alterations caused by the inflammation of the cervical epithelium. When the inflammatory process induced by the STI disrupt the epithelium, high-risk HPV (HR HPV) can penetrate to the basal layer and alter multiple cell activity [17, 19, 20, 31, 32].

Individuals who do not use condoms are at higher risk of infection and reinfection by HPV and other STIs, which also contribute to cervical lesions progression [33, 34], probably leading to the chronic local inflammatory process and intensified immune system stimulation [12, 19, 35]. In contrast, the constant use of condoms is associated with a lower risk of HPV infection and regression of cervical lesions rates, as it allows the immune system to act and repair the damaged tissue, preventing the progression of the lesion. [36].

The analysis of HPV genotyping demonstrated that no association between the specific kinds of HPV and TV was found, though these data must be analyzed with care, since the number of HPV indeterminates was high. In addition, it was possible to verify that not having HPV infection decreases the chance of having TV by 0.272 times, which strengthened the hypothesis of a possible cooperation between both microorganisms, contributing to cellular microenvironment changes.

This study confirms the correlation between TV and HPV among women who sought to undergo cancer screening. Since the participants were evaluated in a cross-sectional study design, we could not identify the causal relationship between these infectious agents. Although physiologically plausible, the mechanism(s) by which an association between TV infection, cervicitis, and vaginal infections in the increased risk of squamous intraepithelial lesions and/or cervical intraepithelial neoplasia a prospective cohort study would be adequate to assess the linkage between TV/HPV coinfections and the development of precursor cancer lesions.

## Conclusion

TV infection was associated with HPV infection of the cervix as well as with cervical cytological abnormalities. The scale and prevalence of co-infections in our study population justify the necessity of attention by public health services and demonstrate the importance of condoms and the frequency in which female sex workers undergo oncotic cytology examinations. Further studies could reveal the mechanisms by which these two organisms interact at the cellular level and how these shared behavioral risks act for the progression of precancer cervical lesions.

## Data Availability

The datasets used and/or analysed during the current study are available from the corresponding author on reasonable request.

## Abbreviations

AIR: Australian Immunization Register
CEP-UFMA: Research Ethics Committee of the Federal University of Maranhão
DNA: deoxyribonucleic acid
ESF: family health strategy
USA: United States of America
HIV: human immunodeficiency virus
HPV: human papillomavirus
IARC: International Cancer Research Agency
STI: sexually transmitted infection
MS: Ministry of Health
WHO: World Health Organization
PCR: polymerase chain reaction
SUS: unified health system
FICF: informed consent form
UBS: basic health units
TV: *Trichomonas vaginalis*
WHO: World Health Organization
